# The Taste of Emotion: Metaphoric Association Between Taste Words and Emotion/Emotion-Laden Words

**DOI:** 10.3389/fpsyg.2020.00986

**Published:** 2020-06-05

**Authors:** Yanyun Zhou, Chi-Shing Tse

**Affiliations:** ^1^Department of Educational Psychology, The Chinese University of Hong Kong, Hong Kong, China; ^2^Centre for Learning Sciences and Technologies, The Chinese University of Hong Kong, Hong Kong, China

**Keywords:** taste, emotion, emotion-laden words, emotion words, conceptual metaphor, embodied cognition

## Abstract

According to the Conceptual Metaphor Theory, abstract concepts can be metaphorically associated with more concrete, physically embodied concepts, such as gustatory experience. Studies on taste–emotion metaphoric association reported that people associate love with sweet, jealousy with sour and bitter, and sadness with bitter. However, few studies have systematically examined the metaphoric association between taste and words referred to emotion (e.g., “sad”) or emotion-laden concepts (e.g., “funeral”). In the current four studies (total *N* = 357), we examined this metaphoric association by having participants come up with a taste word when reading an emotion and emotion-laden word (Study 1—explicit association of taste words-to-emotion/emotion-laden words), come up with an emotion word when reading taste words (Study 2—explicit association of emotion words-to-taste words), rate the association between taste words and basic or non-basic emotion words (Study 3), and rate the association between taste words and a more expanded pool of emotion/emotion-laden words (Study 4). Results showed that sweet was mostly associated with positive emotion and emotion-laden words, whereas bitter, followed by sour and spicy, was mostly associated with negative emotion and emotion-laden words. The bidirectionality of taste–emotion metaphoric association was supported by our dataset. The implications of these findings on the Conceptual Metaphor Theory and embodied cognition are discussed.

## Introduction

Metaphor, a figure of speech in linguistics, is used to describe a concept by another apparently unrelated concept, e.g., “*Jealous is sour/bitter*” (Yu, 1998). However, according to the Conceptual Metaphor Theory (e.g., Lakoff and Johnson, 1980; Landau et al., 2010), metaphors are not only a linguistic phenomenon, but can also reflect how abstract concepts are associated with more concrete, physically embodied concepts. In the present study, we examine how emotion is metaphorically associated with taste. Specifically, we refer the “taste–emotion association” to the association between taste words and emotional words. We focus on the word association, rather than the association with gustatory experience or induced/measured emotion. Following the definition of Sutton and Altarriba (2016), emotional words refer to both emotion words (i.e., words for emotion state, e.g., “sad”) and emotion-laden words (i.e., words with emotional connotation, e.g., “tear”).

In English and Chinese, people can use taste (as the source) to describe emotion (as the target). Bitter taste may refer to unhappy feeling, e.g., “*bitter blow*” and “*苦水* (*bitter water*),” whereas sweet taste (e.g., *sweetie*) may express love among lovers and pleasant emotion, e.g., “*甜到入心 (I feel sweet in heart)*.” Words with opposite tastes can refer to complicated emotion, e.g., “*bitter sweet*” to express mixed feelings of happiness and sadness, and “*甜酸苦辣 (sweet, sour, bitter, spicy)*” to refer to experience of joy and sadness in life. Other than directly describing the state of emotion, tastes can be associated with words with emotional connotation (i.e., emotion-laden words, see Sutton and Altarriba, 2016), e.g., “*甜頭 (sweetener, referring to benefit)*,” “*甜言蜜語 (sweet and honey words, referring to complimentary and flattering words)*,” “*苦盡甘來 (sweet are the fruits of labor)*,” referring to the turning point when adversity is replaced by prosperity. Taste-related words can also be used to describe emotion, e.g., “*吃醋*” *(having vinegar, referring to jealous feeling)*, or “*dry-swallow for soothing or slobber dripping*” to refer to physiological responses to something being preferred, and “*disgust*” to express our dislike to something.

Despite the common use of taste words to express emotion in daily language, relatively few studies have investigated their metaphoric association. Most of those focused on sweet and bitter tastes as source concepts (e.g., Eskine et al., 2011; Meier et al., 2012; Hellmann et al., 2013; Ren et al., 2015). Relative to the control condition (e.g., salty taste induced by consuming potato chips), sweet taste, as induced by having participants consume cookies, led them to evaluate a hypothetical relationship to be more favorable and show more interest to begin a relationship with a potential partner (e.g., Ren et al., 2015). After tasting sweet, compared with bitter beverage, participants exhibited a more lenient tendency toward people who take revenge on others (e.g., Hellmann et al., 2013). Exposing participants with sweet taste led them to infer themselves to be more agreeable and boosted their motive to help others (e.g., Meier et al., 2012), but inducing participants with bitter taste increased their disgust ratings toward moral transgression (e.g., Eskine et al., 2011). Survival motivation could be embodied in bitter taste: Participants performed better for survival-related words in lexical decision task or memory task after drinking bitter lotus root or chewing bitter lotus root relative to control condition (e.g., drinking plain water) (e.g., Chen and Chang, 2012). All these suggest that sweet is associated with favorable interpersonal characteristics like prosocial personality. Priming participants with specific taste could influence their emotion perception, interpersonal judgments, and even behavioral intents.

There was also evidence for the opposite direction of the taste–emotion metaphoric association. Feeling gratitude, rather than pride, as triggered by reading description of kind actions, led participants to show higher preference for consuming sweet food (e.g., Meier et al., 2012; Schlosser, 2015). While participants tended to judge a hypothetical person to be more agreeable based on his/her preference for sweet food, agreeable people also showed a higher preference for sweet food (Meier et al., 2012). Relative to reading events that were irrelevant to morality, reading moral transgression (or morally virtue) events induced participants to rate bland water with more disgusting (or delicious) taste (e.g., Eskine et al., 2012). Participants’ preference for bitter taste was positively correlated with their antisocial personality and negatively associated with their agreeableness (e.g., Sagioglou and Greitemeyer, 2016). In short, the metaphoric association between taste and emotion may be bidirectional, consistent with some (e.g., brightness–emotion in Huang et al., 2018) but not the other conceptual metaphors (e.g., spatial–emotion in Huang and Tse, 2015).

It is important to examine the taste–emotion association at the word level. Lee and Schwarz (2012) suggested that a conceptual metaphor involves both linguistic and psychological consequences, which are not necessarily corresponding with each other (Murphy, 1996, 1997). The former indicated how a concept was talked about in language, and the latter indicated people’s feeling, action, and reasoning based on the concept. Since conceptual metaphors were originally explained by Lakoff and Johnson (1999) in linguistics, in the present study, we aim at finding out evidence for the taste–emotion association at the word level. However, there was only one published work directly related to the taste word–emotion word metaphoric association. Chan et al. (2013) examined the metaphoric associations among two emotions (love and jealousy) and three tastes (sweet, bitter, and sour). In Experiment 1A, they had participants rate the association between taste words and emotion words on a 7-point Likert scale (1 = not at all associated; 7 = highly associated) (Two additional taste words, “salty and spicy,” and three additional emotion words, “passion, sadness, and betrayal,” were also included to mask the research purpose). Results showed that participants associated “love” with sweet taste more strongly than bitter or sour taste and associated “jealousy” with sour and bitter tastes more strongly than sweet taste. There was no difference on the rating between bitter and sour tastes with respect to “jealousy.” In Experiment 1B, participants generated at least two taste words to each of the five emotion words (love, jealousy, sadness, betrayal, and passion). Again, “sadness,” “betrayal,” and “passion” were included to mask the research purpose. Results showed that 80.4% of participants first come up sweet in response to love, and 60.8% and 28.4% of participants first come up sour and bitter in response to “jealousy,” respectively. Despite not their focus, their data revealed other taste–emotion metaphoric associations: when the first and second responses were counted, “passion” was associated with sweet (63.7%) and spicy tastes (52.0%), “sadness” was associated with bitter (72.5%) and sour tastes (53.9%), and “betrayal” was associated with bitter taste (84.3%) and sour taste (53.9%). These findings provided preliminary evidence for the taste–emotion metaphoric association of two specific emotions, love and jealousy. However, in contrast to the taste–emotion metaphoric association, there was relatively weak semantic (i.e., out of context) association between emotion and taste words, as reported in Nelson et al. (2004) free association norm: spicy–good, sweet–good, sweet–kind, and sour–bad. Besides, no study has examined the taste–emotion metaphoric association using emotion-laden words. Hence, in the current work, we conducted four studies, using different tasks and with both emotion and emotion-laden words, to further investigate the taste–emotion metaphoric association at the word level.

We adapted previous studies’ paradigms in the current work. In Sutton and Altarriba’s (2016) color–emotion association task, participants were instructed to produce the first color that comes to mind for 390 words that varied in valence and concreteness. They found that red color being associated more with negative emotion and emotion-laden words, whereas yellow color was more associated with positive words for both emotion and emotion-laden words. In Palmer et al. (2013) rating task, they had participants rate the association between emotion words (e.g., happy, sad) and music or color on a −100 to +100 scale. They found emotion was an important mediator for the music–color association. In the current study, we used explicit association task [similar to Sutton and Altarriba’s (2016) one but with taste-to-emotion and emotion-to-taste directions] and association rating task to obtain convergent evidence for the taste–emotion metaphoric association.

Compared with color–emotion association that was intensely examined in the literature (e.g., Elliot, 2015; Sutton and Altarriba, 2016), taste, despite being another important sensation and its being metaphorically associated with emotion, has received much less attention. In the current work, our main goal was to develop a norm for taste–emotion metaphoric association, and based on this database, we explored whether the database could test some premises of the Conceptual Metaphor Theory (e.g., Lakoff and Johnson, 1980), such as bidirectionality. In Studies 1 and 2, we used the explicit association task to norm the data for emotion/emotion-laden word-to-taste word and taste word-to-emotion word metaphoric associations, respectively. In Studies 3 and 4, we had participants rate the metaphoric association across different pairs of taste words and emotional words (i.e., both emotion and emotion-laden words).

## Study 1—Explicit Association of Emotion/Emotion-Laden Word-To-Taste Word

### Methods

#### Participants

One hundred and two participants [67 female; *M* age = 19.93 years (SD = 2.04, range = 17–29); five left-handed] were recruited to participate in exchange of 100 HKD (∼13 USD). In all studies reported in this article, participants were Cantonese-speaking undergraduate or postgraduate students from the Chinese University of Hong Kong (CUHK), had normal gustation and with normal or corrected-to-normal vision, were able to input responses in traditional Chinese characters in the task, and provided informed consent prior to the experiment. All studies were approved by CUHK Survey and Behavioral Research Ethics Committee. No participant participated in more than one study reported in this article.

#### Materials, Design, and Procedure

At the beginning, we translated all 1,034 words in Affective Norms for English Words (ANEW) (Bradley and Lang, 1999) to Chinese. The two authors of this article and one research assistant, two of whom are locals in Hong Kong, checked the translation and excluded 13 words. The translations for six of these eliminated words overlapped with other words in Chinese, e.g., both “bunny” and “rabbit” mean 兔子 in Chinese, so we only included “rabbit.” Two of them (quart and rattle) might not be familiar to Hong Kong students, and the translations of the remaining five eliminated words refer to tastes in Chinese, e.g., “anguished, luscious, and sour.” The remaining 1,021 words, as well as “envy,” which is not in ANEW, were included in the current study.

Following the classification scheme of Sutton and Altarriba (2016) (i.e., words with valence rating lower or equal to 4 being categorized as negative words, words with valence rating higher or equal to 7 being classified as positive words; valence was rated on a 9-point scale, with 1 indicating extremely negative and 9 indicating extremely positive), 237 positive words (37 positive emotion words and 200 positive emotion-laden words), 342 negative words (62 negative emotion words and 280 negative emotion-laden words), and 443 neutral words were presented in the explicit association task. Despite being listed out, the data of one positive emotion word “safe” and one negative emotion word “cane” were not included in data analyses due to inappropriate translation. Since the present study focused on taste–emotion metaphoric association, only 236 positive words (36 positive emotion words and 200 positive emotion-laden words) and 341 negative words (62 negative emotion words and 279 negative emotion-laden words) were included in the final analyses (see Supplementary Appendix 1 for the word list).

Since we quantified the valence measure of our Chinese word stimuli based on ANEW norm, which was based on English word stimuli and English native speakers, we performed some analyses to check the validity of this valence measure for our Chinese population. Specifically, we compared the valence measure of the ANEW norm with the valence measure of the norm that was developed in our previous study (Huang et al., 2018). The measure in this latter norm, which consists of 696 Chinese words, was based on Hong Kong students, i.e., the same population as in the current study. Across two norms, 568 words were overlapped, so we could examine whether the valence measures of these 568 words (i.e., 81.6% of words in the current norm) from the ANEW (i.e., based on native English speakers) and Huang et al.’s (2018) norm (i.e., based on Hong Kong students) would be consistent. The correlation analyses revealed that the correlation between the valence measure in Huang et al.’s (2018) norm and the valence measure in ANEW was very strong (*r* = 0.875). Similar analyses on the arousal also showed a moderate-to-strong correlation (*r* = 0.649). Given the very strong correlation between the valence measure of ANEW and the valence measure of Huang et al.’s (2018) norm in 81.6% of the words included in the current norm, we consider it appropriate to refer to the valence measure of the ANEW in the current study. Besides, the analyses of the current norm based on ANEW valence measure would be more informative for future researchers who would want to select words from our norm for their experiments being conducted in English-speaking participants.

Participants completed the explicit association task given as an online questionnaire in two 50-min sessions in 2 successive days. They did that on computers in separate cubicles in groups of 2–3 in a quiet laboratory. The instruction and words were presented in both Chinese and English. The 1,022 words were randomly divided into two sets, which were given in the two sessions, respectively. Within each session, words were presented in two blocks, and participants were allowed to take a short break between the blocks. The words assigned to the two sessions and to the two blocks within each session were counterbalanced between participants. The presentation order of the words within each block was freshly randomized for each participant. The participants were verbally reminded to type their answers in Chinese and fill in one taste for each word. If they thought of more than one, they were instructed to put down the first one that comes to mind. While there was no time limit, participants were told not to spend too much time on any specific words. The instruction was: “*For each concept, please think of the first taste that comes to mind and type your answer in the field. For example, if you see the concept ‘difficulty,’ you might think of ‘bitter,’ so you should type ‘bitter’ in the field. If you cannot think of a taste, or you don’t think that the given concept is associated with any taste, you should type ‘no.’ If you are not sure whether the answer you think of is a taste or a smell, the way to distinguish them is: If it could be sensed by tongue, it is a taste. If it is sensed by nose, it is a smell. You need to fill in the taste. If you have any questions, please ask the experimenter at any time.*”

### Results

The “no” responses, which were given when participants could not think of any taste for the words, were included in our analyses because they suggest the absence of taste-related association for particular words. Supplementary Appendix 2 lists the taste response and their frequency, percentage for each word of each word type.

Results were summarized in Table 1: For negative emotion words, the most frequent response was bitter (56.73%), followed by sour (14.79%) and spicy (13.85%). For negative emotion-laden words, the most frequent response was also bitter (46.99%), followed by spicy (16.19%) and “no response” (i.e., unable to come up with any taste, 15.01%). The most frequent response for positive emotion words was sweet (78.27%), followed by “no response” (7.84%) and spicy (6.48%). For positive emotion-laden words, the most frequent response was also sweet (64.89%), followed by “no response” (15.81%) and salty (5.44%).

**TABLE 1:** Summary of taste responses and their frequency and percentage for four kinds of emotional words.

Taste (Chinese)	Taste (English)	Frequency	Percentage
**Negative emotion words**			
苦	Bitter	3,587	56.73
酸	Sour	935	14.79
辣	Spicy	876	13.85
No	No	442	6.99
鹹	Salty	304	4.81
澀	Astringent	55	0.87
甜	Sweet	50	0.79
甘	Sweet and bitter	20	0.32
淡	Bland	17	0.27
辛	Pungent	15	0.24
腥	Bloody	10	0.16
醱	Taste by fermentation	3	0.05
香	Savory	2	0.03
辛辣	Pungent	1	0.02
鮮	Umami	1	0.02
酸甜	Sweet and sour	1	0.02
麻	Numbing taste	1	0.02
苦澀	Bitter and astringent	1	0.02
鹹苦	Salty and bitter	1	0.02
爽	Refreshing	1	0.02
緑	Green	1	Invalid
Total (valid)		6,323	100
**Negative emotion-laden words**			
苦	Bitter	13,352	46.99
辣	Spicy	4,599	16.19
No	No	4,264	15.01
酸	Sour	2,935	10.33
鹹	Salty	1,804	6.35
甜	Sweet	789	2.78
腥	Bloody	189	0.67
澀	Astringent	118	0.42
辛	Pungent	96	0.34
甘	Sweet and bitter	70	0.25
淡	Bland	69	0.24
血腥	Bloody	49	0.17
鮮	Umami	15	0.05
腥	Fishy	11	0.04
麻	Numbing taste	10	0.04
爽	Refreshing	8	0.03
香	Savory	8	0.03
濃	Rich-flavored	5	0.02
油膩	Greasy	4	0.01
酸辣	Sour and spicy	3	0.01
酸甜	Sweet and sour	3	0.01
血	Bloody	2	0.01
苦澀	Bitter and astringent	2	0.01
酦	Taste by fermentation	2	0.01
冷	Cold	1	0.00
苦辣	Bitter and spicy	1	0.00
鮮鹹	Umami and salty	1	0.00
餿	Soured	1	0.00
薄荷	Taste of mint	1	0.00
甘苦	Sweet and bitter	1	0.00
臭	Stink	25	Invalid
腥	Rank-smelling	17	Invalid
銹	Rusty	1	Invalid
吱	Squeak	1	Invalid
痛	Painful	1	Invalid
Total (valid)		28,413	100
**Positive emotion words**			
甜	Sweet	2,874	78.27
No	No	288	7.84
辣	Spicy	238	6.48
酸	Sour	86	2.34
鹹	Salty	60	1.63
苦	Bitter	50	1.36
甘	Sweet and bitter	34	0.93
鮮	Umami	14	0.38
淡	Bland	11	0.30
酸甜	Sweet and sour	4	0.11
爽	Refreshing	3	0.08
辛	Pungent	3	0.08
腥	Bloody	2	0.05
濃	Rich-flavored	2	0.05
辛辣	Pungent	1	0.03
香	Savory	1	0.03
甜辣	Sweet and spicy	1	0.03
Total (valid)		3,672	100
**Positive emotion-laden words**			
甜	Sweet	13,234	64.89
No	No	3,225	15.81
鹹	Salty	1,109	5.44
苦	Bitter	840	4.12
酸	Sour	717	3.52
辣	Spicy	705	3.46
甘	Sweet and bitter	240	1.18
鮮	Umami	77	0.38
淡	Bland	71	0.35
澀	Astringent	55	0.27
腥	Bloody	36	0.18
爽	Refreshing	29	0.14
辛	Pungent	16	0.08
酸甜	Sweet and sour	12	0.06
香	Savory	6	0.03
濃	Rich-flavored	6	0.03
甜辣	Sweet and spicy	4	0.02
甘苦	Sweet and bitter	2	0.01
濃烈	Rich-flavored	2	0.01
甘甜	Sweet and bitter	1	0.00
油膩	Greasy	1	0.00
甜鹹	Sweet and salty	1	0.00
蜜糖	Honey flavor	1	0.00
冰涼	Very cold	1	0.00
薄荷	Taste of mint	1	0.00
麻	Numbing taste	1	0.00
冷	Cold	1	0.00
生	Fresh	1	0.00
臭	Sink	2	Invalid
Nature	Nature	1	Invalid
笨	Stupid	1	Invalid
他	He	1	Invalid
Total (valid)		20,395	100

*Invalid responses have not been counted for the result on percentage of taste responses. The response “腥(xing)” were translated into three kinds of meanings: fishy, bloody, rank-smelling depend on the stimuli words. For example, “腥(xing)” was translated into “fishy” for “dinner”; “bloody” for “assassin”; “rank-smelling” for “manure”.*

#### Reliability

To test the reliability, we followed Sutton and Altarriba (2016) and randomly divided 102 participants into two groups (Samples A and B). Then, we computed the total set size (TSS) and mean set size (MSS) for each word for each of the two groups. TSS is the total amount of different taste responses for each word by all participants (frequency of the response could be one or more than one) in the group. MSS is the total amount of different responses for each word by two or more participants (frequency for the response is two or more than two) in the group. Since there might be idiosyncratic response for the words, we consider MSS a more representative indicator. See Supplementary Appendix 3 for the taste response for each word in each subsample. Pearson correlation coefficients of TSS/MSS between the two groups were significant for all word types (all *p*s < 0.01). For negative emotion words, *r* = 0.528 for MSS, *r* = 0.326 for TSS. For negative emotion-laden words, *r* = 0.442 for MSS, *r* = 0.217 for TSS. For positive emotion words, *r* = 0.805 for MSS, *r* = 0.501 for TSS. For positive emotion-laden words, *r* = 0.700 for MSS, *r* = 0.592 for TSS. Similar to Sutton and Altarriba (2016), we obtained high correlations between the percentages of overlapping taste responses to each word from two samples in each word type, negative emotion words, *r* = 0.96 (SD = 0.06), negative emotion-laden words, *r* = 0.94 (SD = 0.09), positive emotion words, *r* = 0.98 (SD = 0.06), and positive emotion-laden words, *r* = 0.96 (SD = 0.07).

For negative emotion and negative emotion-laden words, the correlations between MSS of two groups were lower than correlation for positive emotion and positive emotion-laden words. This might be attributed to the possibility that among the five common tastes (sweet, bitter, sour, spicy, and salty), positive words were mostly associated with sweet taste, whereas negative words could be associated with more than one of the five tastes, thereby leading to more diverse taste responses to negative words. In Table 1, there was a large proportion of sweet response for positive emotion (78.27%) and positive emotion-laden words (64.89%), whereas none of the other taste-related responses reached 10%. In contrast, for negative emotion words and negative emotion-laden words, other than bitter taste (56.73% for negative emotion words and 46.99% for negative emotion-laden words), >10% of participants’ responses were related to spicy and sour tastes, suggesting that participants’ taste-related responses were indeed more diverse for negative words than for positive words. To further verify this idea, we examined the data of TSS and MSS of the whole sample for each word type, see Table 2 for the results on descriptive statistics. Using independent *t*-test, we tested the difference between negative and positive emotion words on TSS and MSS and showed that for emotion words, there was significant difference on TSS, *t*(96) = 2.713, *p* < 0.01, and on MSS, *t*(96) = 4.484, *p* < 0.001. The mean of MSS was higher for negative words than for positive words. For emotion-laden words, there was no significant difference between negative and positive words on TSS, *t*(477) = −0.164, *p* = 0.870, but the mean of MSS was significantly higher for negative words than for positive words, *t*(477) = 3.506, *p* < 0.01. Thus, the lower consistency for TSS and MSS for negative words from two groups could be due to participants’ more diverse taste-related responses for negative words than for positive words.

**TABLE 2:** Means and standard deviation for total set size (TSS) and mean set size (MSS) for each of the four kinds of emotional words for the whole sample.

	Negative emotion words	Negative emotion laden words	Positive emotion words	Positive emotion laden words
TSS	6.84 (1.19)	7.29 (1.28)	5.92 (1.83)	7.31 (1.69)
MSS	5.14 (0.86)	5.59 (0.85)	3.75 (1.75)	5.17 (1.51)

## Study 2—Explicit Association of Taste Word-To-Emotion Word

### Methods

#### Participants

One hundred and five participants [75 female, *M* age = 19.45 years (SD = 1.71, range = 16–25), six left-handed] were recruited to participate in exchange of 5 HKD (∼0.64 USD). Data of five additional participants, who did not follow the instruction, were excluded.

#### Materials, Design, and Procedure

Participants were instructed to come up with an emotion word that first come into mind for each of the five tastes, sour, sweet, bitter, spicy, and salty (in Study 1, these were the most frequent taste responses. Umami, one of the five common tastes, was not used because it was rarely provided by participants in Study 1). The presentation order of the taste words was freshly randomized for each participant. All other procedures were the same as those in Study 1. The instruction was: “*For each taste, please think of the first emotion that comes to mind and type your answer in the space. If you cannot think of an emotion, or you don’t think that the given taste is associated with any emotion, you should type ‘no.’ If you have any questions, please ask the experimenter immediately*.”

### Results

Emotion word responses associated with tastes by participants were listed in Supplementary Appendix 4. Despite the variety of participants’ responses, bitter was more associated with “sad” (40.95%) and “agonized” (20.00%), salty was more associated with “no responses” (i.e., unable to come up with any emotion words, 50.96%), sour was more associated with “envy” (25.71%), spicy was more associated with “angry” (35.58%) and “irritated” (12.50%), and sweet was more associated with “happy” (85.71%). To test the bidirectionality of emotion and taste associations, we checked the emotion words that participants generated in Study 2 in norm developed in Study 1. Results showed that “sad” was mostly associated with bitter (75%), “agony” with bitter (84%), “envy” with sour (49%), “angry” with spicy (65%), “irritate” with spicy (65%), and “happy” with sweet (99%). These findings provided preliminary evidence for the bidirectional taste–emotion metaphoric association.

## Study 3—Rating Task for the Association of Taste Word-To-Emotion Word

### Methods

#### Participants

One hundred twenty participants [85 female; *M* age = 19.51 years (SD = 1.61, range = 17–25), four left-handed] were recruited to participate in exchange of 5 HKD (∼0.64 USD).

#### Materials, Design, and Procedure

Participants were asked to rate the strength of association between one taste and each of the 13 emotion words (six basic emotions: anger, fear, disgust, happiness, sadness, and surprise; seven non-basic emotions: anxiety, love, depression, contempt, pride, shame, and envy) (Nummenmaa et al., 2014). In each page of the questionnaire, a taste word was given, and participants were instructed to rate the strength of association between this taste word and each of the 13 emotion words. This design made it easier for participants to compare the strength of association between one taste word and different emotion words. To reduce the carryover influence due to the presentation order of five taste words, we used Latin Square to counterbalance the order of the five taste words between participants. For one taste word, the order of 13 emotion words was randomized. Participants were asked to, based on their first impression, rate the strength of association between taste word and emotion word on a six-point scale (0 = not at all, 1 = very weak, 2 = weak, 3 = moderately, 4 = strong, and 5 = very strong). All other procedures were the same as Study 1. The instruction was: *“There were five pages for this task. On each page, a taste (e.g., sour) is given, you need to rate the strength of association between the taste and each of 13 emotions on a six-point scale (0 to 5). If you don’t think the given taste is associated with any emotion, you could choose ‘0 not at all,’ and if you think the association between the taste and the emotion is very strong, you should choose ‘5 very strong.’ The following is the strength each number indicates, the strength of the association increases from 0 to 5. 0 not at all. 1 very weak. 2 weak. 3 moderately. 4 strong. 5 very strong.”*

### Results

#### Inter-Rater Reliability

The Cronbach’s alpha was 0.989 for all 65 words (i.e., 13 emotions × 5 tastes) across 120 participants.

Table 3A presents the means and standard deviation of the association ratings of 13 emotion words for each taste in a descending order of means. “Envy” was strongly associated with sour (*M* = 3.88, SD = 1.36), both “happiness” and “love” were strongly associated with sweet (*M* = 4.61, SD = 0.74 and *M* = 4.43, SD = 0.86, respectively), both “depression” and “sadness” were strongly associated with bitter (*M* = 4.11, SD = 0.99 and *M* = 4.02, SD = 1.12, respectively), “anger” was strongly associated with spicy (*M* = 4.09, SD = 1.13), and both “sadness” and “disgust” were associated with salty (*M* = 2.32, SD = 1.59; *M* = 2.30, SD = 1.49, respectively), although it was noteworthy that the overall rating was lower for salty than for other tastes. These results were consistent with those reported in Study 2 when an explicit association task was used.

**TABLE 3A:** The means and standard deviation for the rating on taste-emotion associative strength.

Bitter			Salty			Sour			Spicy			Sweet		
Emotion	Mean	SD	Emotion	Mean	SD	Emotion	Mean	SD	Emotion	Mean	SD	Emotion	Mean	SD
Depression	4.11	0.99	Sadness	2.32	1.59	Envy	3.88	1.36	Anger	4.09	1.13	Happiness	4.61	0.74
Sadness	4.02	1.12	Disgust	2.3	1.49	Sadness	2.84	1.34	Surprise	2.74	1.39	Love	4.43	0.86
Anxiety	3.16	1.36	Envy	1.96	1.42	Anxiety	2.53	1.40	Envy	2.33	1.50	Pride	3.18	1.23
Disgust	2.78	1.57	Depression	1.93	1.37	Shame	2.34	1.48	Love	2.05	1.55	Surprise	2.04	1.42
Envy	2.78	1.52	Anxiety	1.89	1.40	Disgust	2.18	1.41	Anxiety	1.98	1.50	Envy	1.09	1.21
Fear	2.63	1.53	Shame	1.82	1.46	Depression	2.18	1.37	Sadness	1.9	1.39	Disgust	0.97	1.18
Shame	2.6	1.52	Contempt	1.63	1.35	Surprise	2.12	1.52	Pride	1.89	1.50	Sadness	0.88	1.16
Anger	1.99	1.42	Fear	1.61	1.33	Contempt	2.06	1.47	Contempt	1.87	1.48	Anxiety	0.77	0.99
Contempt	1.97	1.34	Surprise	1.51	1.42	Fear	1.83	1.32	Happiness	1.84	1.40	Depression	0.63	1.03
Surprise	1.3	1.23	Anger	1.41	1.25	Love	1.58	1.30	Fear	1.72	1.43	Shame	0.58	0.84
Love	0.87	1.06	Happiness	1.21	1.11	Anger	1.48	1.28	Shame	1.23	1.28	Fear	0.49	0.69
Pride	0.8	1.12	Love	1.18	1.11	Happiness	1.27	1.17	Disgust	1.17	1.15	Anger	0.44	0.73
Happiness	0.77	1.05	Pride	1.01	1.14	Pride	0.78	0.97	Depression	1.01	1.16	Contempt	0.43	0.85

We obtained the differences in the ratings of taste–emotion metaphoric association for each emotion word (Table 3B). To statistically compare the differences in the ratings of taste–emotion metaphoric association among five tastes for each emotion, we conducted one-way repeated-measures ANOVA, with the taste being the independent variable, on each of the 13 emotion words. The results are summarized in Table 3C. For each emotion, the strongest taste associations were “anger” –spicy, “anxiety”–bitter, “contempt”–sour, “depression”–bitter, “disgust”–bitter, “envy”–sour, “fear”–bitter, “happiness”–sweet, “love”–sweet, “pride”–sweet, “sadness”–bitter, “shame”–bitter, and “surprise”–spicy.

**TABLE 3B:** The Means and Standard deviation for the associative strength of five tastes for 13 basic emotion.

Anger			Anxiety			Contempt			Depression			Disgust			Envy			Fear		
Taste	Mean	SD	Taste	Mean	SD	Taste	Mean	SD	Taste	Mean	SD	Taste	Mean	SD	Taste	Mean	SD	Taste	Mean	SD
Spicy	4.09	1.13	Bitter	3.16	1.36	Sour	2.06	1.47	Bitter	4.11	0.99	Bitter	2.78	1.57	Sour	3.88	1.36	Bitter	2.63	1.53
Bitter	1.99	1.42	Sour	2.53	1.40	Bitter	1.97	1.34	Sour	2.18	1.37	Salty	2.30	1.49	Bitter	2.78	1.52	Sour	1.83	1.32
Sour	1.48	1.28	Spicy	1.98	1.50	Spicy	1.87	1.48	Salty	1.93	1.37	Sour	2.18	1.41	Spicy	2.33	1.50	Spicy	1.72	1.43
Salty	1.41	1.25	Salty	1.89	1.40	Salty	1.63	1.35	Spicy	1.01	1.16	Spicy	1.17	1.15	Salty	1.96	1.42	Salty	1.61	1.33
Sweet	0.44	0.73	Sweet	0.77	0.99	Sweet	0.43	0.85	Sweet	0.63	1.03	Sweet	0.97	1.18	Sweet	1.09	1.21	Sweet	0.49	0.69

**Happiness**			**Love**			**Pride**			**Sadness**			**Shame**			**Surprise**					
**Taste**	**Mean**	**SD**	**Taste**	**Mean**	**SD**	**Taste**	**Mean**	**SD**	**Taste**	**Mean**	**SD**	**Taste**	**Mean**	**SD**	**Taste**	**Mean**	**SD**			

Sweet	4.61	0.74	Sweet	4.43	0.86	Sweet	3.18	1.23	Bitter	4.02	1.12	Bitter	2.60	1.52	Spicy	2.74	1.39			
Spicy	1.84	1.40	Spicy	2.05	1.55	Spicy	1.89	1.50	Sour	2.84	1.34	Sour	2.34	1.48	Sour	2.12	1.52			
Sour	1.27	1.17	Sour	1.58	1.30	Salty	1.01	1.14	Salty	2.32	1.59	Salty	1.82	1.46	Sweet	2.04	1.42			
Salty	1.21	1.11	Salty	1.18	1.11	Bitter	0.80	1.12	Spicy	1.90	1.39	Spicy	1.23	1.28	Salty	1.51	1.42			
Bitter	0.77	1.05	Bitter	0.87	1.06	Sour	0.78	0.97	Sweet	0.88	1.16	Sweet	0.58	0.84	Bitter	1.30	1.23			

**TABLE 3C:** Results of repeated-measures ANOVA for each of the 13 emotion.

Emotion	F test	Pair-wise analysis
Anger	*F*(4,476) = 211.664, *p* < 0.001, *$\eta_{p}^{2}=0.640$*	Spicy > bitter > sour = salty > sweet
Anxiety	*F*(4,476) = 64.795, *p* < 0.001, *$\eta_{p}^{2}=0.353$*	Bitter > sour > spicy = salty > sweet
Contempt	*F*(4,476) = 42.066, *p* < 0.001, *$\eta_{p}^{2}=0.261$*	Sour = bitter = spicy = salty > sweet
Depression	*F*(4,476) = 187.407, *p* < 0.001, *$\eta_{p}^{2}=0.612$*	Bitter > sour = salty > spicy > sweet
Disgust	*F*(4,476) = 52.370, *p* < 0.001, *$\eta_{p}^{2}=0.306$*	Bitter > salty = sour > spicy = sweet
Envy	*F*(4,476) = 82.161, *p* < 0.001, *$\eta_{p}^{2}=0.408$*	Sour > bitter > spicy > salty > sweet
Fear	*F*(4,476) = 53.629, *p* < 0.001, *$\eta_{p}^{2}=0.311$*	Bitter > sour = spicy = salty > sweet
Happiness	*F*(4,476) = 314.601, *p* < 0.001, *$\eta_{p}^{2}=0.726$*	Sweet > spicy > sour = salty > bitter
Love	*F*(4,476) = 212.524, *p* < 0.001, *$\eta_{p}^{2}=0.641$*	Sweet > spicy > sour > salty > bitter
Pride	*F*(4,476) = 116.559, *p* < 0.001, *$\eta_{p}^{2}=0.495$*	Sweet > spicy > salty = bitter = sour
Sadness	*F*(4,476) = 99.852, *p* < 0.001, *$\eta_{p}^{2}=0.456$*	Bitter > sour > salty > spicy > sweet
Shame	*F*(4,476) = 58.287, *p* < 0.001, *$\eta_{p}^{2}=0.329$*	Bitter = sour > salty > spicy > sweet
Surprise	*F*(4,476) = 26.979, *p* < 0.001, *$\eta_{p}^{2}=0.185$*	Spicy > sour = sweet > salty = bitter

*For Tables 3A,B, the association was rated on a six-point (0–5) scale, with 0 indicated “not at all associated,” and 5 indicated “the association is very strong.” This is also applicable for the following Tables involving association rating. In Table 3C, for “contempt,” differences between sour and salty (p < 0.01), bitter and salty (p < 0.05) were significant. For “pride,” difference between salty and sour was significant (p < 0.05).*

## Study 4—Rating Task for the Association of Emotion/Emotion-Laden Word-To-Taste Word

### Methods

#### Participants

Thirty participants [24 female, *M* age = 19.67 years (SD = 1.81, range = 17–23), all right-handed] were recruited to participate in exchange of 300 HKD (∼38.32 USD). Data from one additional participant, who did not complete the task, were excluded.

#### Materials, Design, and Procedure

The 1,022 emotion words in Study 1 were included. We excluded two words “cane” and “safe” for data analyses because of inappropriate translation. All words were randomly divided into nine sets, each of which was presented in separate online questionnaire. Participants completed all nine sets in three sessions, which were separated by at least 2 h, in 2 or 3 successive days. The presentation orders of online questionnaire were counterbalanced between participants. The presentation order of five tastes for each emotion/emotion-laden word was randomized for each participant. Participants complete the rating task on computers in separate cubicles in groups of 4–10. In the rating task, participants were given an emotion/emotion-laden word on each page of the online questionnaire. They needed to rate, based on their first impression, the association between the word and five tastes (sour, sweet, bitter, spicy, and salty) on a 6-point scale. All other procedures were the same as those in Study 3. The instruction was: “*On each page, a concept (e.g., ‘difficulty’) is given, you need to rate the strength of association between the concept and each of five tastes on a 6-point scale (0 to 5). If you don’t think the given concept is associated with one taste, you could choose ‘0 not at all,’ and if you think the association between the concept and one taste is very strong, you should choose ‘5 very strong.’ The following is the strength each number indicates, the strength of the association increases from 0 to 5. 0 not at all. 1 very weak. 2 weak. 3 moderately. 4 strong. 5 very strong.”*

### Results

#### Inter-Rater Reliability

The Cronbach’s alpha was 0.939 for all the 5,110 rating scores (1,020 words × 5 tastes) across 30 participants. And the Cronbach’s alpha was 0.947 for the 2,885 rating scores (577 words × 5 tastes) for emotion and emotion-laden words across 30 participants.

Similar to Study 1, 577 emotion/emotion-laden words were included in data analyses. Participants rated negative emotion words and negative emotion-laden words most strongly associated with bitter, then followed by sour and spicy (Table 4A). In contrast, sweet was rated more strongly associated with positive emotion and emotion-laden words. This was consistent with Study 1’s finding that bitter and sweet tastes were mostly given in response to negative and positive words, respectively. As shown in Table 4A, mean association ratings of negative emotion word–bitter, negative emotion-laden word–bitter, positive emotion word–sweet, and positive emotion-laden word–sweet were all higher than 3.0, providing further support for the sweet–positive/bitter–negative metaphoric association. The mean association ratings of sour and spicy with negative emotion words were moderate (2.59 and 2.07, respectively), whereas none of the tastes was as strongly associated with positive emotion words as sweet (all below 1.66), suggesting that negative words were associated with bitter and less so with sour and spicy, but positive words were associated with sweet only. We used repeated-measures ANOVAs to test the difference on the mean association ratings among the five tastes in each of the four emotion word types (see Table 4B for the results). In Supplementary Appendix 5, the rating score of five tastes for each word were presented in a descending order, and the results of repeated-measures analyses for the difference between the association ratings with five tastes, as well as the pairwise analysis if the difference was significant. In Supplementary Appendix 6, we selected the words from Supplementary Appendix 5 which was associated with one taste significantly stronger than each of other four tastes.

**TABLE 4A:** Means and standard deviation of each taste for the four kinds of emotional words (sorted in descending order by means).

Negative emotion words	Negative emotion laden words	Positive emotion words	Positive emotion laden words
Bitter 3.35 (0.60)	Bitter 3.17 (0.69)	Sweet 3.64 (0.74)	Sweet 3.24 (0.75)
Sour 2.59 (0.44)	Sour 2.28 (0.50)	Spicy 1.66 (0.82)	Bitter 1.58 (0.64)
Spicy 2.07 (0.82)	Spicy 2.13 (0.78)	Sour 1.47 (0.41)	Sour 1.58 (0.44)
Salty 1.69 (0.22)	Salty 1.71 (0.38)	Salty 1.26 (0.34)	Spicy 1.54 (0.69)
Sweet 0.72 (0.29)	Sweet 0.79 (0.46)	Bitter 1.14 (0.48)	Salty 1.43 (0.56)

**TABLE 4B:** Results of repeated-measures ANOVA on five taste responses for four kinds of emotional words.

Negative emotion words	Negative emotion laden words	Positive emotion words	Positive emotion laden words
*F*(4,244) = 210.192, *p* < 0.001, *$\eta_{p}^{2}=0.775$*	*F*(4,1112) = 640.095, *p* < 0.001, *$\eta_{p}^{2}=0.697$*	*F*(4,140) = 120.257, *p* < 0.001, *$\eta_{p}^{2}=0.775$*	*F*(4,796) = 349.070, *p* < 0.001, *$\eta_{p}^{2}=0.637$*
Bitter > sour > spicy > salty > sweet	Bitter > sour > spicy > salty > sweet	Sweet > spicy = sour > salty = bitter	Sweet > bitter = sour = spicy > salty

To reexamine the potential bidirectionality of taste–emotion association, we computed the correlation between taste–emotion metaphoric association ratings obtained in Study 3 (taste word-to-emotion word) and in Study 4 (emotional word-to-taste word) (Table 5). Pearson correlation coefficients for the association ratings of the 65 words (13 emotion × 5 tastes) were very high (*r* = 0.902, *p* < 0.001), suggesting the high consistency in the association ratings for the taste–emotion metaphoric association from either direction.

**TABLE 5:** Comparison on means for the association strength of five tastes and 13 basic emotion from study 3 and study 4.

Anger			Anxiety			Contempt			Depression			Disgust		
Taste	Study 3	Study 4	Taste	Study 3	Study 4 (anxious)	Taste	Study 3	Study 4	Taste	Study 3	Study 4	Taste	Study 3	Study 4 (disgusted)
Spicy	4.09	4.00	Bitter	3.16	3.13	Sour	2.06	2.73	Bitter	4.11	4.20	Bitter	2.78	3.43
Bitter	1.99	3.13	Sour	2.53	2.57	Bitter	1.97	2.87	Sour	2.18	2.67	Salty	2.30	1.97
Sour	1.48	2.10	Spicy	1.98	1.93	Spicy	1.87	1.87	Salty	1.93	1.53	Sour	2.18	3.40
Salty	1.41	1.70	Salty	1.89	1.83	Salty	1.63	1.33	Spicy	1.01	1.53	Spicy	1.17	1.23
Sweet	0.44	0.53	Sweet	0.77	0.80	Sweet	0.43	0.50	Sweet	0.63	0.37	Sweet	0.97	0.47

**Envy**			**Fear**			**Happiness**			**Love**					
**Taste**	**Study 3**	**Study 4**	**Taste**	**Study 3**	**Study 4**	**Taste**	**Study 3**	**Study 4 (happy)**	**Taste**	**Study 3**	**Study 4**			

Sour	3.88	3.70	Bitter	2.63	3.13	Sweet	4.61	4.53	Sweet	4.43	4.63			
Bitter	2.78	3.20	Sour	1.83	2.17	Spicy	1.84	1.47	Spicy	2.05	2.53			
Spicy	2.33	2.40	Spicy	1.72	2.40	Sour	1.27	1.20	Sour	1.58	2.73			
Salty	1.96	1.53	Salty	1.61	1.53	Salty	1.21	1.13	Salty	1.18	2.07			
Sweet	1.09	0.67	Sweet	0.49	0.40	Bitter	0.77	0.90	Bitter	0.87	2.57			

**Pride**			**Sadness**			**Shame**			**Surprise**					
**Taste**	**Study 3**	**Study 4**	**Taste**	**Study 3**	**Study 4 (sad)**	**Taste**	**Study 3**	**Study 4 (shamed)**	**Taste**	**Study 3**	**Study 4 (surprised)**			

Sweet	3.18	2.63	Bitter	4.02	3.90	Bitter	2.60	2.73	Spicy	2.74	2.80			
Spicy	1.89	1.57	Sour	2.84	3.10	Sour	2.34	2.77	Sour	2.12	2.23			
Salty	1.01	1.43	Salty	2.32	2.23	Salty	1.82	1.77	Sweet	2.04	2.47			
Bitter	0.80	1.43	Spicy	1.90	1.50	Spicy	1.23	1.37	Salty	1.51	1.63			
Sour	0.78	1.40	Sweet	0.88	0.50	Sweet	0.58	0.80	Bitter	1.30	1.63			

*Some emotion words were of the different pattern (noun and adjective) in studies 3 and 4, e.g. for “anxiety” in the study 3, the paired word in study 4 was “anxious.”*

## General Discussion

### Conceptual Metaphoric Association Between Taste and Emotion

Based on a large pool of emotion/emotion-laden words and taste words, in four studies, we systematically investigated the metaphoric association between taste and emotion using explicit association tasks with both taste-to-emotion and emotion-to-taste directions and taste–emotion metaphoric association rating tasks. In this investigation, we have developed norms for associations between taste words and emotional words. Analysis based on these norms showed that sweet was associated with positive emotion/emotion-laden words, bitter, followed by sour and spicy, was associated with negative emotion/emotion-laden words. Specifically, sweet was associated with “happiness” and “love,” bitter with “sad” and “agonized,” sour with “envy,” spicy with “angry” and “irritated,” and salty with “no” response (i.e., not associated with any emotion). The data of the norm also provided potential evidence for bidirectionality of the taste–emotion metaphoric association. In the following discussion, when our findings are quoted, *frequency* refers to response frequency in the explicit association task and *M* and SD refer to mean and standard deviation for association ratings.

Our findings provided a direct evidence for the conceptual metaphor association. On one hand, the word associations shown in the norm are consistent with those reported in previous studies on taste-related conceptual metaphor. For example, we found that “disgust” was associated with bitter (frequency = 50%, *M* = 3.43, SD = 1.17, being significantly higher than those of other four tastes), supporting this association reported in previous works (e.g., Eskine et al., 2011). Moreover, previous research found being gratitude, relative to being pride, promoted participants’ preference for sweet food consuming (e.g., Meier et al., 2012; Schlosser, 2015). In the present study, the ratings of “grateful”–sweet association (*M* = 3.97, SD = 1.07) was significantly stronger than those of “pride”–sweet association [*M* = 2.63, SD = 1.59, *t*(29) = 3.92, *p* < 0.001] or “proud”–sweet association [*M* = 3.00, SD = 1.60, *t*(29) = 2.99, *p* < 0.01], which provided a more direct comparison of strength between “gratitude”–sweet and “pride/proud”–sweet associations. Furthermore, previous study found that in romantic relationship, “acceptance” was sweet and “rejection” was bitter (e.g., DeWall and Bushman, 2011), while in the present study, “rejected” was associated with bitter (frequency = 69%, *M* = 3.57, SD = 1.50, which was significantly higher than those of the other four tastes), and “acceptance,” “hug,” “kiss,” and “wedding” were all moderately-to-strongly associated with sweet [frequency = 51%, 92%, 93%, and 94%; *M* = 2.67 (SD = 1.58), 4.17 (SD = 1.15), 4.60 (SD = 0.67), and 4.33 (SD = 0.84), respectively, and all were significantly higher than the associations with the other four tastes]. On the other hand, our norm could provide insight for researchers to find out more conceptual metaphor associations, which should then be tested in experiments in future studies.

### Bidirectionality of Taste–Emotion Metaphoric Association

In four studies, we found consistent metaphoric association in both taste-to-emotion and emotion-to-taste directions, such as bitter–“sad,” sour–“envy,” spicy–“angry,” and sweet–“happy,” in the explicit association task. As indicated by the association ratings, the taste-to-emotion and emotion-to-taste associations were highly correlated (*r* = 0.902, *p* < 0.001), suggesting the consistent taste–emotion association from either direction. According to the Conceptual Metaphor Theory (Lakoff and Johnson, 1980; Lakoff and Johnson, 1999), abstract concepts (target concept) was presented and understood through the more concrete perceptual and sensorimotor experience (source concept), but not the other way around. Thus, the direction of metaphoric association should be concrete-to-abstract, not abstract-to-concrete. However, some previous studies challenged this view by showing that the metaphoric association could be activated in both concrete-to-abstract and abstract-to-concrete (e.g., Meier and Robinson, 2004; Schubert, 2005; Jostmann et al., 2009; Schneider et al., 2011; Lee and Schwarz, 2012; Huang et al., 2018). For example, Lee and Schwarz (2012) found that the metaphoric association of fishy smell and suspicion was bidirectional. Priming participants with fishy smell elicited suspicion and reduced cooperation in a trust-based exchange, and socially induced suspension also improved the correct percentage on labeling the fishy smell. Neural coactivation mechanism might account for the bidirectionality. Neural connections can be developed in the process when people experience the cross-domain correlation between abstract and concrete concepts since early life. This connection enables the coactivation of brain areas for both conception when either one of them is activated. This repeated experience built up the basis of conceptual structure (e.g., Lakoff and Johnson, 1999; Lakoff, 2008). This view was supported by neuroimaging findings related to taste perception (e.g., Grabenhorst et al., 2008; Yamamoto, 2008; Ren et al., 2015). Positive emotion such as love and sweet taste shared similar neural substrates [anterior cingulate cortex (ACC)] (e.g., Ren et al., 2015), whereas amygdala that responds for rewarding could be activated by sweet taste (e.g., Yamamoto, 2008). Hence, people learn the taste–emotion metaphoric association since early age and the conceptual structure of this association is built up deeply in our brain, thus showing its bidirectionality when either one is activated. Schneider et al. (2011) suggested that embodied effect might explain the bidirectionality of one association. Given that abstract concepts are grounded in concrete concepts, the bidirectionality of the association is attributed to the co-occurrence of the abstract concept and concrete bodily state. As Landau et al. (2010) pointed out, the inconsistent findings on bidirectionality made it important for researchers to find out “whether, when and how metaphors were bidirectional.” The present study may provide a preliminary evidence for the bidirectionality, but it is noteworthy that experiments involving the manipulations of gustatory experience and emotion should be conducted to provide stronger evidence for or against the bidirectionality of metaphoric associations.

### Is Taste–Emotion Metaphoric Association Language-Dependent?

The generality of conceptual metaphoric association between taste and emotion across Chinese and English languages was observed as we obtained findings, which were based on Chinese words, being consistent with those reported by Chan et al. (2013), which were based on English words (Table 6). The taste–emotion metaphoric association was quite consistent across two studies. Both studies found “love” was strongly associated with sweet, “passion” also with sweet, “jealousy” with sour, “sadness” with bitter, and “betray” also with bitter. It is noteworthy that only five English emotion words and native English speakers (as participants) were used in Chan et al. (2013). In our work, we generalized their findings by including a much larger pool of words in another language (Chinese) and with native Chinese speakers as participants. These were not too surprising because the taste–emotion metaphoric association could be found in both English and Chinese texts. The “good taste–subjective good feelings” and “bad taste–subjective bad feelings” mapping might be originated from British 18th century in Europe when taste was often used to indicate esthetic appreciation (Vainik, 2018). In the Bible, the relationship between taste and emotion (affection) is often cited, e.g., “*you, men, love your wives and be not bitter towards them.*” Bitter and sweet tastes are put as opposites in two extremes as the way good and evil did, e.g., in the Bible (Js5:20) “*Cursed are those who give the name of good to evil, and of evil to what is good: who make light dark, and dark light: who make bitter sweet, and sweet bitter!*” Similarly, in Chinese, taste was often used to indicate emotion, especially in the songs and literacy works. The song, “Coffee, Tea or Me, I love you,” begins with “sadness and happiness, bitter and sweet” (悲傷歡喜，苦澀甜蜜), whereas in the poem “Laolao Pavilion” (勞勞亭) by Libai, a famous poet in ancient China, bitter was used to indicate the heartbrokenness caused by farewell. “天下傷心處，勞勞送客亭。春風知別苦，不遺柳條青。” [A most heartbreaking place in the world, Is the Laolao Pavilion of Parting. Knowing the bitterness of such occasions; The spring breeze lets not the willows turn green (Willow twigs were plucked and given as a farewell gift in ancient times)] (Wen et al., 1995). However, as the studies on the taste–emotion metaphoric association were rather limited, the historical and cultural roots of taste–emotion metaphoric mapping across different cultures await further investigation.

**TABLE 6:** Comparison of results from Chan et al. (2013) and the present study.

		Free association task				Association rating task	
		Chan et al. (2013) (Study 1B)		The present study (Study 1)		Chan et al. (2013) (Study 1A)	The present study (Study 4)
					
**Words**	**Tastes**	**Frequency/total**	**Percentage**	**Frequency/total**	**Percentage**	**Rating**	**Rating**
Love	Sweet	82/102	80.39	86/102	84.31	5.41	4.63
	Bitter			3/102	2.94	3.38	2.57
	Sour			5/102	4.90	2.76	2.73
Jealousy	Sour	62/102	60.78	50/102	49.02	5.32	3.7
	Bitter	29/102	28.43	28/102	27.45	4.97	3.13
	Sweet			1/102	0.98	1.54	0.93
Passion	Sweet	65/143	45.45	49/102	48.04		3.27
	Spicy	53/143	37.06	39/102	38.24		3.9
	Sour	11/143	7.69	2/102	1.96		1.77
	Salty	11/143	7.69	3/102	2.94		1.77
	Bitter	3/143	2.10	1/102	0.98		1.13
Sad	Bitter	74/150	49.33	77/102	75.49		3.9
	Sour	48/150	32.00	14/102	13.73		3.1
	Salty	23/150	15.33	6/102	5.88		2.23
	Sweet	4/150	2.67	1/102	0.98		0.5
	Spicy	1/150	0.67	2/102	1.96		1.5
Betray	Bitter	86/159	54.09	62/102	60.78		4.07
	Sour	55/159	34.59	18/102	17.65		3.07
	Spicy	16/159	10.06	16/102	15.69		2.43
	Salty	2/159	1.26	1/102	0.98		1.77
	Sweet	0/159	0.00	0/102	0.00		0.37

*There was a bit difference on the procedure of the experimental task between Chan et al. (2013) and the present study: For free association task: In Chan et al. (2013), they had participants generate at least two tastes for each emotion word. In the present study, we had participants think of one taste first come into mind for each word. For association rating task: In Chan et al. (2013), participants rate the associative strength on a 7-point scale, with 1 indicated not at all associated, 7 indicated highly associated. While in the present study, participants rate the associative strength on a 6-point scale, with 0 indicated not at all associated, 1 indicate very weak association, and 5 indicated very strong association. There was also difference on data coding of the presented results for free association task: In Chan et al. (2013), for the words “love” and “jealousy” in study 1B, frequency and percentage were coded based on the first taste response provided by 102 participants, the same as in our study. However, for the words “passion, sad, betray,” frequency and percentage were coded based on the valid responses as both first and second responses for each word provided by 102 participants. In the present study, frequency and percentage were all coded based on the first response (valid) to each word by 102 participants.*

### Implications of the Current Findings on the Embodied Cognition

The present findings could shed light on the embodied cognition. According to the embodied cognition theory (e.g., Lakoff and Johnson, 1999; Anderson, 2003), what we experienced bodily could influence what we process in mind. People acquire knowledge of concepts by interacting with the physical world (e.g., by seeing, touching, and tasting) *via* their sensorimotor system. For example, priming physical warmth increased the likelihood of judging a stranger to be friendlier (social warmth) (e.g., Williams and Bargh, 2008). Holding heavier clipboards made people tend to judge the issue or person being reviewed to be more important (e.g., Jostmann et al., 2009; Ackerman et al., 2010). Touching rough surface led people to judge social interaction to be less coordinated (e.g., Ackerman et al., 2010). Related to the present study, taste also had embodied effect on psychological states, for example, sweet taste boosted peoples’ motivation to help others (Meier et al., 2012). In contrast, bitter taste induced emotional and moral disgust (e.g., Chapman et al., 2009). According to Lee and Schwarz (2012), sometimes the embodied effect could be mediated by metaphorical associations, for example, the embodied effect of fishiness and social suspicion, and it could be dated back to the preliminary theory of Lakoff and Johnson (1999), repeated co-occurrences between abstract states like emotion and concrete bodily sensations like gustation. The current findings provide evidence for the taste-related embodiment by showing the strong taste–emotion metaphoric association and other taste-related associations. The embodiment of taste and psychological states and activities (e.g., emotion) might probably be built up in our early life and then be strengthened *via* repeated pairings of this association throughout our lives, making it easier for certain conceptions to be activated than the other ones. For example, Chan et al. (2013) stated that love might be embodied in sweet taste in the infant period, since breast or bottled milk tasted sweet and being fed was a signal to babies as being loved and cared, so that the association between sweet sensation and love feelings can be developed. Future studies should explore further on the embodiment of actual taste, e.g., whether the taste of a chocolate could alter participants’ self-reported emotion.

Before concluding the current study, one could argue that the taste–emotion metaphoric association might merely result from taste diagnosticity; that is, the degree to which a taste is representative of an object or is associated with an object (e.g., sugar and honey are sweet; balsam and lotus seed are bitter). To examine the taste diagnosticity, we checked the semantic association of our word stimuli in University of South Florida free association norm (Nelson et al., 2004). It is possible that some words might be higher in taste diagnosticity than the other words, particularly words with high concreteness in emotion-laden words (e.g., sugar, candy). If taste diagnosticity played an important role, emotion words would be strongly associated with one certain taste. We found that among 1,022 words, only 19 of our positive emotion-laden words were associated with a specific taste, with mean associative strength of 0.094, ranging from 0.014 to 0.451. Among them, the stronger sweet-related association was with “honey,” 0.451, then “sugar,” 0.433, “candy,” 0.336, “cute,” 0.113, and “chocolate,” 0.101. The associative strength for other words with sweet was all lower than 0.10. Hence, for most of the words in our studies, there was no strong semantic association with specific taste, we could exclude the possibility that the taste diagnosticity was largely responsible for the tastes normed for our emotion or emotion-laden words.

One of the limitations of our study was that we used emotional words and taste words to test the taste–emotion association, and the word–word association could only provide preliminary evidence for the bidirectionality of taste–emotion association. This should be further investigated by including other modality and measurement, e.g., induced gustation by chocolate on emotion changes, which was investigated in our lab (e.g., Zhou and Tse, under review). In that study, we tested the impact of chocolates tastes (manipulated by different cocoa levels) on measured emotion, and the impact of induced emotion on taste perception, which could provide a more direct test for the directionality of taste–emotion association. The other limitation was that, in the present study, we did not examine the role that the arousal of emotional words might play in the taste–emotion metaphoric association. While it is true that both valence and arousal are important for emotion, it is noteworthy that previous works on emotional word association did not take into account word arousal (e.g., Nummenmaa et al., 2014; Sutton and Altarriba, 2016). To our knowledge, no taste–emotion word association studies have taken into account the word “arousal.” Nevertheless, it is important for future researchers to explore whether the word “arousal” may moderate the taste–emotion metaphoric association. Another limitation was that there were more female (70.3%) than male in our participants. To our knowledge, there has not been any evidence for the gender difference on the association between taste words and emotion words. Indeed, this gender ratio was quite common in previous works that reported the association norms. In Sutton and Altarriba’s (2016) emotion–color association study, their participants were predominately female (75 out of 94, i.e., 79.8%). Similarly, in Gilman et al.’s (2017) norm study on emotional film clips, their participants were also mostly female (596 out of 784, i.e., 76%). Thus, we do not consider our gender ratio would be particularly problematic. Nevertheless, the extent to which participants’ gender might moderate the association between taste words and emotion words should be further investigated in future research.

## Conclusion

In our everyday life, taste is most commonly related to food, and it could be perceived everywhere every day. Previous research reported that taste could be related to prosocial behaviors (e.g., Meier et al., 2012), aggressive behaviors (e.g., Hellmann et al., 2013), moral decisions (e.g., Eskine et al., 2011), and intimate relationships (e.g., Ren et al., 2015). In four studies with different tasks (explicit association and association rating), the present study tested of taste–emotion metaphoric association by a large dataset. Analyses on this dataset provided support for the bidirectionality of conceptual metaphor, contrary to the view of Lakoff and Johnson’s (1999) Conceptual Metaphor Theory. It is hoped that our normed dataset will provide experimental materials to further our understanding on the taste-related conceptual metaphors (e.g., grateful–sweet association) and embodied cognition (e.g., the influence of gustatory experience on emotion).

## Data Availability Statement

All datasets generated for this study are included in the article/Supplementary Material.

## Ethics Statement

The studies involving human participants were reviewed and approved by the CUHK Survey and Behavioral Research Ethics Committee. The participants provided their written informed consent to participate in this study.

## Author Contributions

YZ and C-ST contributed to the study design and wrote the manuscript. YZ performed the data collection and analyses. Both authors approved the final version of the manuscript for submission.

## Conflict of Interest

The authors declare that the research was conducted in the absence of any commercial or financial relationships that could be construed as a potential conflict of interest.
